# Correlation between nuclear factor κB activity and pulmonary artery pressure in a rat high pulmonary blood flow model

**DOI:** 10.3892/etm.2014.2121

**Published:** 2014-12-08

**Authors:** JIE YANG, XIAO-XIAO YU, ABDUHAER ABULAITI, JIAN-CHUN FEI

**Affiliations:** 1 Department of Pediatrics, Qilu Hospital of Shandong University, Jinan, Shandong 250012, P.R. China; 2 Department of Pediatrics, The First Affiliated Hospital of Xinjiang Medical University, Urumqi, Xinjiang 830054, P.R. China; 3 Department of Anesthesiology, Qilu Hospital of Shandong University, Jinan, Shandong 250012, P.R. China

**Keywords:** nuclear factor κB, pyrrolidine dithiocarbamate, vascular endothelial cells, pulmonary vascular remodeling

## Abstract

The aim of the present study was to investigate the correlation between nuclear factor-κB (NF-κB) activity and pulmonary artery pressure in the pulmonary artery endothelial cells of high pulmonary blood flow rat models. A total of 50 four-week-old male Wistar rats were randomly divided into four groups: Surgery shunt group (Tn, n=15); surgery + pyrollidine dithiocarbamate (PDTC) administration group (Ti, n=15); sham control group (Co, n=10) and negative control group (Cn, n=10). The 30 rats of the Ti and Tn groups underwent carotid artery-external jugular vein anastomosis; the 15 rats in the Ti group were injected with PDTC intraperitoneally 1 h prior to surgery for a two-week continuous infusion. After 12 weeks of feeding *ad libitum*, right ventricular systolic pressure and NF-κB activity in the pulmonary artery endothelial cells of the rats were measured. The NF-κB activity of the Tn group was significantly higher than that of the Cn group (P<0.01) and the NF-κB activity of the Ti group was lower than that of the Cn group (P<0.01); however, no significant difference was observed between the Co and Cn groups. The increased activity of NF-κB was an important factor in the pulmonary vasoconstriction and structural remodeling of rats with high pulmonary blood flow.

## Introduction

Left-to-right shunt congenital heart disease (CHD) causes pulmonary vascular wall endothelial cell damage by powerful stretch stimulation and high shear stress due to the impact of a large flow of blood through the pulmonary circulation (1). Endothelial injury destroys the endothelial barrier function and the muscle-endothelial connection between endothelial and smooth muscle cells. It also destroys the balance between vascular endothelial cells and vasoactive substances produced by the pulmonary circulation, as well as the regulation of endothelial cell and smooth muscle cell formation, thus contributing to the proliferation, hypertrophy and disorganization of the pulmonary vascular smooth muscle cells and causing pulmonary vascular structural remodeling. It has been demonstrated that the nuclear factor-κB (NF-κB) activation pathway exists in the vascular endothelium, smooth muscle cells and cardiac myocytes (2,3). In the present study, the method of establishing animal models of high pulmonary blood flow was used to measure the NF-κB activity of the experimental and control groups, as well as pulmonary artery pressure and NF-κB activity changes subsequent to the administration of an NF-κB inhibitor [pyrollidine dithiocarbamate (PDTC)]. The aim of the study was to determine the correlation between NF-κB activation and pulmonary hypertension in a high blood flow model.

## Materials and methods

### Animals

Fifty four-week-old male Wistar rats (purchased from the Experimental Animal Center of Shandong University School of Medicine, Jinan, China) with an average weight of 120 g were randomly divided into four groups by computer: Surgical shunt group (Tn, n=15); surgery + PDTC administration group (Ti, n=15); sham surgery group (Co, n=10) and negative control group (Cn, n=10). The present study was carried out in strict accordance with the recommendations in the Guide for the Care and Use of Laboratory Animals of the National Institutes of Health. The animal use protocol was reviewed and approved by the Institutional Animal Care and Use Committee of Shandong University.

### High pulmonary blood flow modeling

The 30 rats from the Tn and Ti groups underwent carotid artery-external jugular vein anastomosis to establish a left-to-right shunt pulmonary hypertension model. Fifteen rats in the Ti group were injected intraperitoneally with PDTC 1 h prior to surgery with a dose of 120 mg/kg/day for two weeks of continuous infusion. With the exception of the common carotid artery-jugular venous anastomosis, the rats in the Co group underwent identical surgical procedures to the experimental groups. The rats underwent 12 weeks of *ad libitum* under specific pathogen-free conditions.

### Measurement of pulmonary artery pressure

Chloral hydrate (10%, 0.3 ml/100 g; Sigma-Aldrich, St. Louis, MO, USA) was used to anesthetize the rats. A 3F right heart catheterization was performed by inserting the catheter into the right ventricle of the rats via the right external jugular vein (in perspective). The right ventricular systolic pressure was recorded through a pressure sensor pipeline (Omega, Stamford, CT, USA), and was assumed to be theoretically equivalent to the pulmonary artery systolic pressure (PASP).

### Measurement of cardiac morphological indicators

The heart was cut at the place where the right ventricle and the ventricular septum meet. The weight of the right and the left ventricles were obtained using an electronic balance (Adventurer AR1530; Mettler Toledo, Shanghai, China), and the right to left ventricle plus septum weight ratio was calculated.

### Measurement of pulmonary vascular index

Lung tissue sections were stained with hematoxylin and eosin and the morphology of the pulmonary artery was observed under a light microscope (Olympus BX41; Olympus Corporation, Tokyo, Japan). The external diameter (ED) and media wall thickness of the middle artery (50–200 μm in diameter) was measured and at least 10 middle pulmonary arteries were measured. The mean percentage of the media wall thickness in the artery was calculated using the following formula: (MT% = 2×MT/ED×100%), where MT is the medial wall thickness.

### Determination of NF-κB activity

Following thoracotomy, the pulmonary artery was rinsed with phosphate-buffered saline (PBS), extracted and then placed in 4°C PBS (ph=7.4) with 2 × 10^5^ U/l penicillin and 200 mg/l streptomycin, and the pulmonary artery endothelial cells were isolated and cultured. The medium was aspirated and discarded prior to the cells being washed with 5 ml ice-cold PBS/phosphatase inhibitor. This rinsing fluid was subsequently discarded and 3 ml ice-cold PBS/phosphatase inhibitor was added to the cells. The cells were then carefully transferred from the culture flask to a pre-cooled 15-ml cone-shaped bottle using a cell scraper. The cell suspension was centrifuged at 4°C and 14,000 × g for 5 min, the supernatant was discarded and the cell pellet was placed on ice. A total of 500 *μ*l 1× hypotonic buffer (Sigma-Aldrich) was added to the cell pellet and the cells were aspirated to form a suspension. The cell suspension was transferred to a pre-cooled microfuge tube and incubated on ice for 15 min. A total of 25 *μ*l detergent was added and the suspension was vortexed on the highest setting for 10 sec and centrifuged at 4°C by microcentrifuge at 14,000 × g for 30 sec. The upper suspension was then transferred to a pre-cooled microcentrifuge tube (−80°C) and stored for use.

The precipitate was fully resuspended in 50 *μ*l lysis buffer and vortexed for 10 sec. The suspension was then placed on ice in a shaker (VWR International, Radnor, PA, USA) at 4,200 × g to incubate for 30 min. Following high-speed vortexing for 30 sec and centrifugation at 4°C and 14,000 × g for 10 min, the supernatant was transferred to pre-cooled microcentrifuge tubes to store at −80°C. The supernatant contained the desired nucleoprotein. An NF-κB gene DNA probe was prepared with the following sequences: Upper strand, 5′-AGT TGA GGG GAC TTT CCC AGG C-3′; lower strand, 3′-TCA ACT CCC CTG AAA GGG TCC G-5′. The 5′ and 3′ ends were labeled with biotin digoxin. An electrophoretic mobility shift assay (digoxin-labeled NF-κB kit; Roche Diagnostics, Indianapolis, IN, USA) was used to measure NF-κB activity. The procedures were performed according to the manufacturer’s instructions and the results were obtained by computer image analysis. Optical density represented the NF-κB activity.

### Statistical analysis

Data are presented as the mean ± standard deviation. SPSS version 17.0 statistical software (SPSS Inc., Chicago, IL, USA) was used for the Student’s t-test. P<0.05 was considered to indicate a statistically significant difference.

## Results

### General information

During the shunt surgery, one rat in the Tn group died and one in the Ti group. During the observation period, one rat in the Tn group died, one in the Ti group and one in the Co group. When the animals were sacrificed following manometry, one rat in the Ti group was excluded due to a shunt barrier. As a result, the Tn group had 13 rats, the Ti group had 12 rats, the Cn group had 10 rats and the Co group had nine rats. The mean ratio of pulmonary to systolic flow of all shunt rats was 2.32±0.44, indicating that the shunt was smooth and that pulmonary blood flow was significantly increased.

### PASP

The pulmonary artery contraction pressure of the Tn group was significantly higher than that of the Cn group (41.4±2.7 vs. 16.1±3.6 mmHg, P<0.01) while the pulmonary artery contraction pressure of the Ti and Cn groups showed no significant difference (22.5±5.9 vs. 16.1±3.6 mmHg, P>0.05). No significant difference was observed between the Co and Cn groups (15.7±3.1 vs. 16.1±3.6 mmHg, P>0.05) (Table I).

### Cardiac morphological indicators

The ratio of the mass of the right ventricle to that of the left ventricle plus septum was significantly higher in the Tn group than that in the Cn group (0.57±0.07 vs. 0.32±0.03, P<0.01). No significant difference was observed between the Ti and Cn groups (0.37±0.02 vs. 0.32±0.03, P>0.05) or the Co and Cn groups (0.32±0.01 vs. 0.32±0.03, P>0.05) (Table I).

### Pulmonary vascular index

The pulmonary vascular MT% of the Tn group was increased significantly compared with that of the Cn group (55.6±3.3 vs. 13.5±2.1, P<0.01). No significant difference was observed between the Ti and Cn groups (15.8±4.1 vs. 13.5±2.1, P>0.05) or the Co and Cn groups (14.2±1.6 vs. 13.5±2.1, P>0.05) (Table I).

### NF-κB activity

**Figure 1:**
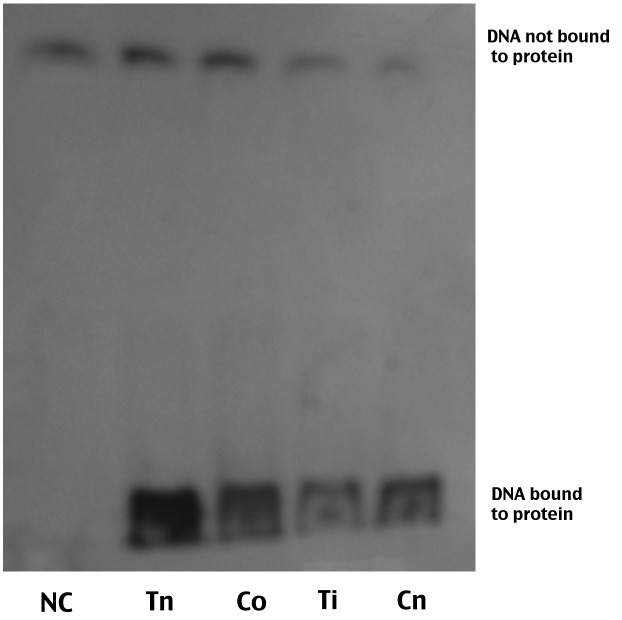
Nuclear factor-κB activity of rat pulmonary artery endothelial cells in the four groups shown by electrophoretic mobility shift assay. From left to right are the negative control (NC), Tn, Co, Ti and Cn groups in turn.

NF-κB activity in the Tn group was significantly higher than that in the Cn group (529±15.1 vs. 298±18.7, P<0.01). The NF-κB activity of the rats in the Ti group was significantly lower than that in the Cn group (228±19.6 vs. 298±18.7, P<0.01). A significant difference was also observed between the Ti and Tn groups (529±15.1 vs. 228±19.6, P<0.01), but no significant difference was found between the Cn and Co groups (314±20.5 vs. 298±18.7, P>0.05) (Table II and Fig 1).

## Discussion

The pathogenesis and treatment of pulmonary hypertension caused by left-to-right shunt CHD is a worldwide clinical concern. Left-to-right shunt CHD results in pulmonary endothelial structural and functional changes due to the pulmonary blood flow, causing significant damage to the vascular endothelium. The endothelial cell damage destroys endothelial barrier functions and muscle-endothelial connections between endothelial cells and smooth muscle cells, as well as undermines the regulatory mechanisms of endothelial cell and smooth muscle cell formation so that vascular smooth muscle cells undergo uncontrollable proliferation (4). In the present study, carotid artery-external jugular vein anastomosis was used to establish the left-to-right shunt animal model, and cardiac catheterization techniques were used to measure the pulmonary artery pressure of the right ventricle. The results showed that the pulmonary artery contraction pressure of the Tn group was significantly higher than that of the Cn group, and this was accompanied by increased right ventricular weight and significant pulmonary vascular structural changes, indicating that the established pulmonary hypertension model was in line with experimental requirements.

Although the pathogenesis of pulmonary hypertension is unknown, evidence suggests that numerous inflammatory cytokines and chemokines play an important role in the course of the disease (5,6). The upregulation of pro-inflammatory genes in response to environmental stimuli has been shown to be coordinated by transcription factors, among which NF-κB has a pivotal role (7). An immunoglobulin κ light-chain gene-enhancer κB sequence (GGG ACT TTC)-specific binding nuclear protein factor was first detected in the nucleus and extracted from B lymphocytes in 1986 and was named NF-κB (8). NF-κB is a nuclear transcription factor with multiple actions. Previous studies showed that, in resting cells, NF-κB combined with the inhibitor I-κB to produce NF-κB in an inactive form in the cytoplasm. When the cells were stimulated by various factors, I-κB rapidly underwent ubiquitination and proteolysis, and NF-κB was activated and rapidly translocated to the nucleus to combine with the κB motif in the promoter regions of target genes, promoting the gene transcription and protein synthesis (9,10). Since then, NF-κB has been found to exist in several cell types, including endothelial, smooth muscle and cardiac pulmonary vascular cells. It plays an important role in the immune response, inflammation and cell-growth control, and is associated with the occurrence and development of cardiovascular diseases (11–14).

The present study confirmed that, in the high-flow model of pulmonary hypertension, NF-κB activity of the shunt group was significantly higher than that of the negative control group; therefore, NF-κB activation was assumed to exist. It can be speculated that high-flow shear stress acted on the vascular endothelium to activate the NF-κB signaling pathway. Subsequent to administering the NF-κB inhibitor PDTC, pulmonary artery pressure was not significantly increased, and NF-κB activity was decreased, which further indicated that NF-κB mediated the development of pulmonary high blood pressure. The studies of Bartman and Hove (15) and Mironov *et al* (16) also found that mechanical interference could regulate the activity and translocation of NF-κB, thus affecting the regulation of gene expression. The study of the cell-stretch injury model additionally found that over-stretch of the cells allowed NF-κB activation, the initiation of inflammatory gene transcription and major cytokine release (17,18). Studies have demonstrated that shear stress increase caused by high pulmonary blood flow can induce the release of endothelial cells, thus promoting the synthesis and secretion of vasoactive substances increasing smooth muscle cell proliferation, such as endothelin, angiotensin, vascular endothelial growth factor, platelet-derived growth factor and vasoactive peptide U-II (19–22), and thereby inhibiting the synthesis and secretion of the cytokines causing smooth muscle cell proliferation, such as prostaglandins, atrial natriuretic peptide, adrenomedullin, nitric oxide and carbon monoxide (23–26). Pulmonary vascular endothelial cells regulate blood quality and the quantity of vasoactive substances via transformation and uptake, pulmonary vascular endothelial cells regulate blood quality and the quantity of vasoactive substances. Under normal physiological conditions, endothelial cells mainly produce various growth inhibitory factors in order to maintain the normal structure of blood vessels; however, in pathological conditions a variety of stimuli can induce the synthesis and secretion of a number of endothelial cell growth factors to promote pulmonary vascular structural remodeling (3,27).

According to the results of the present study, it can be speculated that the high blood flow shear stress acted on the pulmonary artery endothelial cells, activating NF-κB signaling pathways and initiating gene transcription to produce vasoactive mediators, pulmonary vasoconstriction and pulmonary vascular remodeling, and thus leading to pulmonary hypertension. In the present study, the morphology of the heart, pulmonary vascular index and NF-κB activity were obtained after the rats had been sacrificed. Therefore the abovementioned indexes in rats were not tested *in vivo*. However such tests remain to be investigated in future studies using neuroimaging or by obtaining living specimens.

## Figures and Tables

**Table I:** Hemodynamics and vascular morphological parameter changes in the four model groups.

Groups	N	PASP (mmHg)	RV/LV+SP	MT (%)
Tn	13	41.4±2.7^b^	0.57±0.07^b^	55.6±3.3^b^
Ti	12	22.5±5.9^a^	0.37±0.02^a^	15.8±4.1^a^
Cn	10	16.1±3.6	0.32±0.03	13.5±2.1
Co	9	15.7±3.1^a^	0.32±0.01^a^	14.2±1.6^a^

PASP, pulmonary artery systolic pressure; RV/LV+SP, ratio of the mass of the right ventricle to that of the left ventricle plus septum; MT, medial wall thickness.

a P<0.05 and

b P<0.01 compared with Cn group.

**Table II:** Comparisons of NF-κB activity in the four groups.

Groups	N	NF-κB OD value
Cn	10	298±18.7
Co	9	314±20.5^a^
Tn	13	529±15.1^b^,^c^
Ti	12	228±19.6^b^

NF-κB, nuclear factor κB; OD, optical density.

a P<0.05 and

b P<0.01 compared with Cn group;

c P<0.01 compared with Ti group.
